# Epileptic seizures and link to memory processes

**DOI:** 10.3934/Neuroscience.2022007

**Published:** 2022-03-07

**Authors:** Ritwik Das, Artur Luczak

**Affiliations:** Department of Neuroscience, Canadian Centre for Behavioural Neuroscience, University of Lethbridge, Lethbridge, AB, Canada

**Keywords:** epilepsy, memory consolidation, kindling, long-term potentiation, Alzheimer's disease, reflex epilepsy, extinction therapy

## Abstract

Epileptogenesis is a complex and not well understood phenomenon. Here, we explore the hypothesis that epileptogenesis could be “hijacking” normal memory processes, and how this hypothesis may provide new directions for epilepsy treatment. First, we review similarities between the hypersynchronous circuits observed in epilepsy and memory consolidation processes involved in strengthening neuronal connections. Next, we describe the kindling model of seizures and its relation to long-term potentiation model of synaptic plasticity. We also examine how the strengthening of epileptic circuits is facilitated during the physiological slow wave sleep, similarly as episodic memories. Furthermore, we present studies showing that specific memories can directly trigger reflex seizures. The neuronal hypersynchrony in early stages of Alzheimer's disease, and the use of anti-epileptic drugs to improve the cognitive symptoms in this disease also suggests a connection between memory systems and epilepsy. Given the commonalities between memory processes and epilepsy, we propose that therapies for memory disorders might provide new avenues for treatment of epileptic patients.

## 1. Introduction

During sleep and quiet wakefulness, memory patterns are frequently replayed to consolidate memories. For example, patterns of neuronal activity evoked during a behavioral task are later spontaneously replayed when an animal is resting or sleeping [1]. It has been proposed that epileptic activity is “hijacking” those memory consolidation processes, where repetitive memory replay generates aberrant oscillatory network activity [2]. Our recent work provided new evidence for this hypothesis. Using chronic neuronal recordings in epileptic rats, we found that ictal activity patterns were similar to neuronal patterns occurring spontaneously between seizures [3]. This observation suggests that ictal activity could be a “memory pattern” which gets trapped in attractor-like dynamics (B.L. McNaughton personal communication).

Most seizures are spontaneous, meaning that they do not have any clearly identifiable trigger, and can occur at any time, including sleep [4]–[6]. However, in a subset of epileptic patients, spontaneous seizures are preceded by auras: a sensation of particular smell, lights, or certain thoughts [7],[8], which suggest that “internal” triggers of seizures may be potentially identified. Moreover, about 5% of epileptic patients, have reflex seizures which are evoked by specific stimuli [9]–[12]. For example, reflex seizures in some patients may be elicited by flickering lights, certain sounds, or specific activities [13]. A patient may experience both “spontaneous” and “reflex” seizures, suggesting the same underlying mechanisms [12]. It was also reported that performing a specific action (for example, toothbrushing) as well as just thinking about that action can induce seizures [14]. Thus, there is emerging evidence that spontaneous seizures could be a form of reflex seizures where instead of external stimuli, the memory circuits can initiate seizures by activating neuronal patterns representing particular stimuli [15].

In this perspective we present experimental and clinical studies to illustrate the close relationship between epileptogenesis and memory consolidation mechanisms. We begin by describing the interictal events, and how they relate to memory consolidation processes. Next, we describe kindling model used for seizure induction, and its relation to long-term potentiation (LTP), a model for memory formation [16]. We also discuss how cognitive decline in Alzheimer's disease has been related to the development of convulsive pathways [17]. Finally, we review how reflex seizures could be triggered by specific memories, and we propose how memory extinction therapies could provide a novel approach to reduce seizures. The central idea of this perspective is that memory formation processes and epilepsy may be closely related, which could help in developing new therapies.

## 2. Seizure related consolidation and memory related changes during sleep

Seizure related consolidation refers to the changes that occur in the neuronal activity after a seizure epoch, consisting of the reactivation of brain networks associated with the pathology during the subsequent post-ictal period. Seizures induce highly coherent activity in selected neuronal populations. During subsequent sleep, connections between neurons involved in the convulsive episode are strengthened as compared to pre-seizure connection strength [18]. This selective modification of synapses participating in seizures shows similarity to the changes observed after learning during subsequent sleep, where connections between neurons involved in the learned task are also preferentially strengthened [1],[18]. Similarly, activity patterns during inter-ictal spiking (IIS) can be consolidated during the following sleep. [19]. The IISs recorded minutes before the seizure event display similarity in shape and synchrony with “reactivated” IISs during the post-seizure periods including the slow wave sleep period [20]. IIS propagation is seen to be promoted by sleep, with the non-rapid eye movement (NREM) period particularly conducive to greater spike production and propagation [21]–[23]. These studies provide evidence that seizure induced activity can be consolidated in neuronal circuits using similar mechanism as employed by normal learning and memory functions [1].

Moreover, in childhood epilepsy the presence of IISs disturbs the spatiotemporal coupling mechanisms associated with sleep related memory consolidation [24]. The interplay of IIS with slow wave sleep (SWS) was suggested to affect the spindle-ripple interaction responsible for normal information transfer associated with memory [25]–[30]. The interaction of hippocampal IIS with cortical spindles during NREM sleep has been associated with impaired memory consolidation [31]. Studies have shown that IISs disrupt memory and cognition in both animal models [32] and epileptic patients [33]. Recent studies indicate that this is possibly due to intrahippocampal IISs disrupting memory consolidation during sleep involving the hippocampal and cortical circuits [34],[35]. Those results suggest that IISs could be “hijacking” and disturbing normal memory processes [36],[37].

## 3. High frequency oscillations (HFOs)

An important part of the memory consolidation process is sharp wave-ripple (SWR) activity. SWRs are recorded from the hippocampus as large amplitude negative deflections with occasional co-occurrence of short duration fast oscillations called ripples (110–200 Hz) typically during sleep or rest [38]. SWRs reactivate the same sequential neuronal patterns, which were involved before in learning during wakefulness [1]. In epilepsy, brain networks generate pathological HFOs, which are similar to SWRs. Pathological HFOs are around 80 to 500 Hz in frequency and can be further divided into slower 50–250 Hz and fast 250–500 Hz oscillations [39],[40]. The fast oscillations usually originate in the epileptogenic area [39],[40]. The pathological HFOs are also associated with memory impairments [41] and were shown to disrupt the cognitive functionality of the hippocampus, especially when it is part of the epileptic circuit [42]. Although, pathological HFOs may involve distinct subnetworks of neurons as compared to SWRs [43], there is a strong overlap between the mechanisms underlying SWRs and pathological HFOs [44]. This leads to the suggestion that normal physiological processes which are involved in SWR can be “reused” to generate epileptic HFOs [2],[45].

## 4. Neuronal plasticity mechanisms in epilepsy and in memory processes

### 4.1. Kindling and long-term potentiation (LTP)

Kindling is a mechanism by which specific brain regions are sensitized by an external stimulation to generate electrographic epileptiform discharges leading to behavioral seizures [46]. The stimulation, which could be electrical, chemical, optogenetic, or sensory (for example, tactile or auditory) [46]–[50], recruit neurons to become part of the “kindled” circuit. The behavioral seizures occur later when the kindled activity spreads to the motor cortex [51]. This progression of seizures in the animal, from initially just electrographic activity to bilateral tonic-clonic activity with loss of balance, was categorized into five behavioral stages by Ronald J. Racine [52]. The neural changes induced by kindling are usually long lasting [53],[54]. The resemblance of kindling to chronic focal epilepsy in human patients has resulted in using it as a common epilepsy model in animals [55]–[58].

At the molecular level, the N-methyl-D-aspartate (NMDA) receptors, which are involved in synaptic plasticity underlying normal memory processes, also play an important role in producing seizure activity due to kindling. In particular, kindling increases the expression of NMDA receptors in the dentate granule cells, favoring the formation of excitatory circuits that is associated with the increased susceptibility to seizures [59]. As a result of those changes in NMDA receptors, granule cells produce long duration synaptic currents, leading to a burst spiking mode [60],[61]. This increased activity then propagates into the CA3 area of the hippocampus from the dentate gyrus contributing to the developing epileptic circuits [60].

The lasting synaptic changes leading to convulsive behavior are akin to the physiological mechanisms forming memory engrams [53], which is mediated by LTP [62]. LTP increases the synaptic strength that can last for years [63],[64] serving as a basis for memory at the cellular level [65],[66]. The LTP can be experimentally induced by repetitive, high frequency stimulation of afferent connections [67], most widely studied in the Schaffer collaterals and the perforant pathway [68]. At the synaptic level, LTP is typically mediated by NMDA receptors [69], similarly as described above in the kindling model.

### 4.2. Low frequency electrical stimulation (LFS) and long-term depression (LTD)

As opposed to high frequency electrical stimulation used in kindling, low frequency electrical brain stimulation has been shown to reduce epileptic seizures in animal models [70]–[72]. In an in vitro study, LFS of Schaffer collaterals has been shown to reduce the epileptiform activity in a gradual and persistent fashion [73]. Likewise, in a study in young rat pups, 1 Hz LFS was shown to reduce after-discharges as well as behavioral seizures [74]. The main mechanism by which the LFS reduces the response of the stimulated pathways is LTD [75],[76]. LTD is the opposing process to LTP, and it is implicated in the clearing of old memory traces [77],[78]. Similarly, as kindling and LTP, the LFS and LTD is NMDA depended, as it can be blocked by a NMDA receptor antagonist [73]. Thus, cellular plasticity mechanisms involved in memory formation/disintegration (LTP/LTD) are also playing a crucial part in processes inducing/reversing epileptic activity (kindling/LFS).

## 5. Memory impairments and their relation to seizures

Alzheimer's disease which causes severe memory impairments, was also shown to be accompanied by similar network abnormalities and interneuron dysfunction as in epileptic circuits [79],[80]. Alzheimer's disease leads to a hypersynchronous activity which was suggested to accelerate the progression of dementia [81]. Hypersynchronous activity, similar to ictal activity, was observed in both animal models of Alzheimer's disease and in clinical studies [82]–[84]. For instance, imaging studies using fMRI showed hyperactivity in the hippocampus, which was also accompanied by cognitive impairments affecting pattern separation [85]. Alzheimer's patients were also reported to have silent hippocampal seizures and epileptiform spikes during sleep [86]. Memory impairments and increase in the incidence of epilepsy and seizures is also commonly observed in normally aging animals, including humans (≥ 60 years old) [87]–[91]. Importantly, antiepileptic drugs were shown to offer new therapeutic potential for memory impairments in elderly animals and humans [92],[93], which provides strong support for common underlying neuronal circuit changes in epilepsy and in memory impairments.

## 6. Reflex epilepsy—seizures triggered by memories

Reflex epilepsy refers to any syndromic disorder where the seizures are triggered by a specific stimulus, activity, or memory [94]. Wieser's theory of critical mass states that in reflex seizures, a sensory stimulus may trigger a critical amount of cortical tissue which leads to increased activation of epileptogenic neurons causing a seizure [95],[96]. Thus, the specific sensory stimulus may lead to over-activation in specific brain regions which can induce seizures in susceptible patients [97]. Consistently with this theory, it was reported that in patients with generalized seizures, there are regions of hyper-excitability overlapping with regions responsible for encoding sensory stimuli and complex cognitive tasks [98]. Interestingly, even spontaneous ‘thoughts’ could activate the critical mass of epileptogenic neurons, which could provide an explanation of how reflex seizures could be triggered by a memory recall. For example, it was reported that memory from childhood was triggering seizures in a 69-year-old woman [99]. In another example, specific memories of music led to convulsive episodes [100]–[102]. This shows that memories have an ability to activate the epileptic pathways, similarly to sensory stimuli.

Even in a normal brain, the same neuronal population can be activated by external stimuli as well as by internally generated spontaneous neuronal activity. For example, patterns of neuronal population activity which are triggered by sound or tactile stimuli, could be also observed during spontaneous activity in awake, resting animals [103],[104].

Thus, although reflex seizures are only reported in a small percentage of epilepsy patients, they could involve the same mechanisms as spontaneous seizures. For instance, a typical feature during a reflex seizure is the presence of widely synchronized slow waves during the seizures [105]. Similarly, in epileptic patients with spontaneous (non-reflex) seizures, increased slow wave activity was observed as compared to healthy controls [106]–[108]. This suggests common mechanisms underlying spontaneous and reflex seizure. Therefore, we propose that spontaneous seizures could be seen as a special case of reflex seizures, where internally generated activity like memory patterns can initiate seizures similarly to stimulus driven processes.

## 7. Treatment of epileptic disorder by targeting memory reactivation processes?

In the sections above, we provided arguments that epileptic circuits could be formed in a similar way as memory traces, by strengthening selected pathways. Therefore, extinctions treatments used to reduce traumatic memories may also be applicable to weaken aberrant connections involved in seizures [109]. Below we will discuss such methods, which may provide new directions in developing treatments for epileptic patients.

During memory reactivation, patterns of neuronal activity from a previous learning experience are replayed during subsequent sleep or rest period [110]. Reactivation of memories makes them susceptible to modification [111],[112]. Thus, by targeting specific memories during the reconsolidation processes it is possible to weaken those memory traces [113]. For example, reactivated fear memories coupled with protein synthesis inhibitors injected into the amygdala led to amnesia related to a fear inducing stimulus [111]. Similarly, beta-adrenergic receptor blocker propranolol has been shown to be effective in ameliorating post-traumatic stress disorder (PTSD) related memories [114],[115]. Propranolol was shown to also interfere with memory reconsolidation processes when administered after exposure to stressful stimuli in animals [116] and in humans [117]. This is possibly due to the blockade of noradrenergic activity in the amygdala during the reconsolidation process which is responsible for the encoding of emotionally enhanced memories associated with PTSD [118],[119]. Thus, this type of exposure therapy has been proven to be a valuable option for fear memory extinction [120].

Targeting similar mechanisms may also be worth exploring in animal models of epilepsy. Exposure therapy coupled with protein synthesis inhibitors could be the most directly applied to reduce reflex seizures. For instance, Blundell et al. [121], showed that injecting rapamycin after exposure to conditional fear stimuli blocked traumatic memory reconsolidation and decreased the emotional strength of an established traumatic memory. This suggests that epileptic animals could be briefly exposed to a place or task in which they were conditioned to develop reflex seizures, and right after exposure, they could be intraperitoneally injected with rapamycin, which inhibits protein synthesis needed for the memory reconsolidation process. This treatment could be applied once daily over a period of few days. We propose that such treatment could result in reduction of seizures.

The same principles could be probably also applicable to spontaneous seizures. As described in previous sections, connections in epileptic circuits are selectively strengthened in the post-seizure period, by using likely the same mechanisms as memory consolidation processes [18]. Thus, administrating protein synthesis inhibitors right after seizure could block those processes, resulting in weakening connections involved in the epileptic activity. However, the possibility of treating epilepsy with protein synthesis inhibitors should be taken with extreme caution as more animal experiments are needed to establish the safety and efficiency of such approaches.

## 8. Conclusion

In only about two-thirds of patients, seizures can be controlled with medication [122]. This underlines the need for exploring novel options for epilepsy treatment. In this perspective, we present a close relation between memory formation and epileptogenesis. We propose that treatments used to reduce traumatic memories could also provide new options to explore for curtailing seizures.

We described that brain activity in epilepsy is similar to what is observed in the physiological processes associated with memory formation. Activity patterns such as fast ripples are associated with the recurrent neuronal excitation in epilepsy [123] and are involved in memory formation. Moreover, the seizure associated cell ensembles are reactivated during slow wave sleep in a similar fashion as memory patterns after learning of a new task [20]. There is also evidence to suggest that seizure associated with neuronal reactivation and consolidation may lead to a relocation of the epileptogenic focus from the hippocampus to the neocortex in a manner reminiscent of the transfer of memory traces from the hippocampus to the cortex [18]. This suggests that epilepsy may involve the recruitment of the normal physiological memory processes to form epileptic circuits.

Memory extinction therapies have shown promise for disorders like PTSD, in which traumatic memories are specifically reactivated and their subsequent reconsolidation is blocked [124]. Specifically, the neuronal activity is replayed but instead of strengthening, involved synapses are weakened. Thus, the use of targeted memory reactivation to weaken the neuronal circuitry associated with memories or stimuli triggering reflex seizures could lead to a decrease in the ictal episodes [99],[125]. Similarly, administrating memory reconsolidation blockers after a spontaneous seizure may weaken epileptic networks. In future, we plan to combine electrophysiology with behavioral experiments and modeling [126]–[130] to explore those ideas. In summary, we propose that memory extinction treatments should be explored in animal models of epilepsy as it could offer a promising avenue for helping epileptic patients, especially considering that non-invasive extinctions therapies have been proved to be safe in humans.

## References

[1] Wilson MA, McNaughton BL (1994). Reactivation of hippocampal ensemble memories during sleep. Science.

[2] Beenhakker MP, Huguenard JR (2009). Neurons that Fire Together Also Conspire Together: Is Normal Sleep Circuitry Hijacked to Generate Epilepsy?. Neuron.

[3] Neumann AR, Raedt R, Steenland HW (2017). Involvement of fast-spiking cells in ictal sequences during spontaneous seizures in rats with chronic temporal lobe epilepsy. Brain.

[4] Matos G, Tufik S, Scorza FA (2011). Sleep, epilepsy and translational research: What can we learn from the laboratory bench?. Prog Neurobiol.

[5] Karoly PJ, Rao VR, Gregg NM (2021). Cycles in epilepsy. Nat Rev Neurol.

[6] Amengual-Gual M, Sánchez Fernández I, Loddenkemper T (2019). Patterns of epileptic seizure occurrence. Brain Res.

[7] Gupta AK, Jeavons PM, Hughes RC (1983). Aura in temporal lobe epilepsy: clinical and electroencephalographic correlation. J Neurol Neurosur Ps.

[8] Boada C, Grossman S, Dugan P (2020). Aura Semiology as a Predictor of Outcomes Following Epilepsy Surgery (634). Neurology.

[9] Engel J (2001). A Proposed Diagnostic Scheme for People with Epileptic Seizures and with Epilepsy: Report of the ILAE Task Force on Classification and Terminology. Epilepsia.

[10] Engel J (2006). ILAE classification of epilepsy syndromes. Epilepsy Res.

[11] Fisher RS, Cross JH, D'Souza C (2017). Instruction manual for the ILAE 2017 operational classification of seizure types. Epilepsia.

[12] Koutroumanidis M, Panayiotopoulos C (2004). Reflex seizures and reflex epilepsies. Epilepsy in Children, 2E.

[13] Xue LY, Ritaccio AL (2006). Reflex Seizures and Reflex Epilepsy. Am J Electroneurodiagnostic Technol.

[14] Navarro V, Adam C, Petitmengin C (2006). Toothbrush-Thinking Seizures. Epilepsia.

[15] Irmen F, Wehner T, Lemieux L (2015). Do reflex seizures and spontaneous seizures form a continuum?—Triggering factors and possible common mechanisms. Seizure.

[16] Nguyen PV, Abel T, Kandel ER (1994). Requirement of a Critical Period of Transcription for Induction of a Late Phase of LTP. Science.

[17] Palop JJ, Chin J, Roberson ED (2007). Aberrant Excitatory Neuronal Activity and Compensatory Remodeling of Inhibitory Hippocampal Circuits in Mouse Models of Alzheimer's Disease. Neuron.

[18] Bower MR, Stead M, Bower RS (2015). Evidence for Consolidation of Neuronal Assemblies after Seizures in Humans. J Neurosci.

[19] de Curtis M, Avanzini G (2001). Interictal spikes in focal epileptogenesis. Prog Neurobiol.

[20] Bower MR, Kucewicz MT, st. Louis EK (2017). Reactivation of seizure-related changes to interictal spike shape and synchrony during postseizure sleep in patients. Epilepsia.

[21] Del Felice A, Storti SF, Manganotti P (2015). Sleep affects cortical source modularity in temporal lobe epilepsy: A high-density EEG study. Clin Neurophysiol.

[22] Lambert I, Roehri N, Giusiano B (2018). Brain regions and epileptogenicity influence epileptic interictal spike production and propagation during NREM sleep in comparison with wakefulness. Epilepsia.

[23] Sparks FT, Liao Z, Li W (2020). Hippocampal adult-born granule cells drive network activity in a mouse model of chronic temporal lobe epilepsy. Nat Commun.

[24] Georgopoulou V, Spruyt K, Garganis K (2021). Altered Sleep-Related Consolidation and Neurocognitive Comorbidity in CECTS. Front Hum Neurosci.

[25] Halász P, Bódizs R, Ujma PP (2019). Strong relationship between NREM sleep, epilepsy and plastic functions—A conceptual review on the neurophysiology background. Epilepsy Res.

[26] Hahn MA, Heib D, Schabus M (2020). Slow oscillation-spindle coupling predicts enhanced memory formation from childhood to adolescence. eLife.

[27] Stickgold R (2005). Sleep-dependent memory consolidation. Nature.

[28] Buzsáki G (1996). The Hippocampo-Neocortical Dialogue. Cereb Cortex.

[29] McClelland JL, McNaughton B, O'Reilly R (1995). Why there are complementary learning systems in the hippocampus and neocortex: insights from the successes and failures of connectionist models of learning and memory. Psychol Rev.

[30] Squire LR (2004). Memory systems of the brain: A brief history and current perspective. Neurobiol Learn Mem.

[31] Gelinas JN, Khodagholy D, Thesen T (2016). Interictal epileptiform discharges induce hippocampal–cortical coupling in temporal lobe epilepsy. Nat Med.

[32] Kleen JK, Scott RC, Holmes GL (2010). Hippocampal interictal spikes disrupt cognition in rats. Ann Neurol.

[33] Kleen JK, Scott RC, Holmes GL (2013). Hippocampal interictal epileptiform activity disrupts cognition in humans. Neurology.

[34] Lambert I, Tramoni-Negre E, Lagarde S (2020). Hippocampal Interictal Spikes during Sleep Impact Long-Term Memory Consolidation. Ann Neurol.

[35] Lambert I, Tramoni-Negre E, Lagarde S (2021). Accelerated long-term forgetting in focal epilepsy: Do interictal spikes during sleep matter?. Epilepsia.

[36] Maharathi B, Wlodarski R, Bagla S (2019). Interictal spike connectivity in human epileptic neocortex. Clin Neurophysiol.

[37] Arbune AA, Meritam Larsen P, Wüstenhagen S (2021). Modulation in time of the interictal spiking pattern related to epileptic seizures. Clin Neurophysiol.

[38] Buzsáki G (2015). Hippocampal sharp wave-ripple: A cognitive biomarker for episodic memory and planning. Hippocampus.

[39] Jacobs J, Zijlmans M, Zelmann R (2010). High-frequency electroencephalographic oscillations correlate with outcome of epilepsy surgery. Ann Neurol.

[40] Jacobs J, LeVan P, Chander R (2008). Interictal high-frequency oscillations (80–500 Hz) are an indicator of seizure onset areas independent of spikes in the human epileptic brain. Epilepsia.

[41] Jacobs J, Banks S, Zelmann R (2016). Spontaneous ripples in the hippocampus correlate with epileptogenicity and not memory function in patients with refractory epilepsy. Epilepsy Behav.

[42] Liu S, Parvizi J (2019). Cognitive refractory state caused by spontaneous epileptic high-frequency oscillations in the human brain. Sci Transl Med.

[43] Ewell LA, Fischer KB, Leibold C (2019). The impact of pathological high-frequency oscillations on hippocampal network activity in rats with chronic epilepsy. eLife.

[44] Karlócai MR, Kohus Z, Káli S (2014). Physiological sharp wave-ripples and interictal events in vitro: what's the difference?. Brain.

[45] Augusto R, Mendes V, Zacharias LR (2019). Hijacking of hippocampal-cortical oscillatory coupling during sleep in temporal lobe epilepsy.

[46] Teskey GC (2020). Kindling. Oxford Research Encyclopedia of Psychology.

[47] Goddard G v (1967). Development of Epileptic Seizures through Brain Stimulation at Low Intensity. Nature.

[48] Marescaux C, Vergnes M, Kiesmann M (1987). Kindling of audiogenic seizures in Wistar rats: An EEG study. Exp Neurol.

[49] Cela E, McFarlan AR, Chung AJ (2019). An Optogenetic Kindling Model of Neocortical Epilepsy. Sci Rep.

[50] Shimada T, Yamagata K (2018). Pentylenetetrazole-induced kindling mouse model. J Vis Exp.

[51] McIntyre DC, Poulter MO, Gilby K (2002). Kindling: some old and some new. Epilepsy Res.

[52] Racine RJ (1972). Modification of seizure activity by electrical stimulation: II. Motor seizure. Electroencephalography Clin Neurophysiol.

[53] Goddard G v, Douglas RM (1975). Does the engram of kindling model the engram of normal long term memory?. Can J Neurol Sci.

[54] Goddard G v, McIntyre DC, Leech CK (1969). A permanent change in brain function resulting from daily electrical stimulation. Exp Neurol.

[55] Kundap UP, Paudel YN, Kumari Y (2019). Embelin prevents seizure and associated cognitive impairments in a pentylenetetrazole-induced kindling zebrafish model. Front Pharmacol.

[56] Metcalf CS, Huff J, Thomson KE (2019). Evaluation of antiseizure drug efficacy and tolerability in the rat lamotrigine-resistant amygdala kindling model. Epilepsia Open.

[57] Wada JA (1977). Pharmacological Prophylaxis in the Kindling Model of Epilepsy. Arch Neurol.

[58] McNamara JO (1989). Development of New Pharmacological Agents for Epilepsy: Lessons from the Kindling Model. Epilepsia.

[59] Mody I, Heinemann U (1987). NMDA receptors of dentate gyrus granule cells participate in synaptic transmission following kindling. Nature.

[60] Lynch M, Sayin Ü, Golarai G (2000). NMDA Receptor-Dependent Plasticity of Granule Cell Spiking in the Dentate Gyrus of Normal and Epileptic Rats. J Neurophysiol.

[61] Dalby NO, Mody I (2003). Activation of NMDA Receptors in Rat Dentate Gyrus Granule Cells by Spontaneous and Evoked Transmitter Release. J Neurophysiol.

[62] Bliss TVP, Collingridge GL, Morris RGM (2018). Long-term potentiation in the hippocampus: discovery, mechanisms and function. Neuroforum.

[63] Citri A, Malenka RC (2008). Synaptic Plasticity: Multiple Forms, Functions, and Mechanisms. Neuropsychopharmacology.

[64] Malenka RC, Nicoll RA (1999). Long-Term Potentiation--A Decade of Progress?. Science.

[65] Abraham WC, Jones OD, Glanzman DL (2019). Is plasticity of synapses the mechanism of long-term memory storage?. npj Sci Learn.

[66] Bliss TVP, Collingridge GL (1993). A synaptic model of memory: long-term potentiation in the hippocampus. Nature.

[67] Lømo T (2003). The discovery of long-term potentiation. Philos T Roy Soc B.

[68] Nicoll RA (2017). A Brief History of Long-Term Potentiation. Neuron.

[69] Kauer JA, Malenka RC, Nicoll RA (1988). A persistent postsynaptic modification mediates long-term potentiation in the hippocampus. Neuron.

[70] Ruan Y, Xu C, Lan J (2020). Low-frequency Stimulation at the Subiculum is Anti-convulsant and Anti-drug-resistant in a Mouse Model of Lamotrigine-resistant Temporal Lobe Epilepsy. Neurosci Bull.

[71] Mihály I, Orbán-Kis K, Gáll Z (2020). Amygdala low-frequency stimulation reduces pathological phase-amplitude coupling in the pilocarpine model of epilepsy. Brain Sci.

[72] Paschen E, Elgueta C, Heining K (2020). Hippocampal low-frequency stimulation prevents seizure generation in a mouse model of mesial temporal lobe epilepsy. eLife.

[73] Albensi BC, Ata G, Schmidt E (2004). Activation of long-term synaptic plasticity causes suppression of epileptiform activity in rat hippocampal slices. Brain Res.

[74] Velíšek L, Velíšková J, Stanton PK (2002). Low-frequency stimulation of the kindling focus delays basolateral amygdala kindling in immature rats. Neurosci Lett.

[75] Wagner JJ, Alger BE (1996). Homosynaptic LTD and depotentiation: Do they differ in name only?. Hippocampus.

[76] Chapman KB, Yousef TA, Foster A (2021). Mechanisms for the Clinical Utility of Low-Frequency Stimulation in Neuromodulation of the Dorsal Root Ganglion. Neuromodulation: Technology at the Neural Interface.

[77] Nicholls RE, Alarcon JM, Malleret G (2008). Transgenic Mice Lacking NMDAR-Dependent LTD Exhibit Deficits in Behavioral Flexibility. Neuron.

[78] Malleret G, Alarcon JM, Martel G (2010). Bidirectional Regulation of Hippocampal Long-Term Synaptic Plasticity and Its Influence on Opposing Forms of Memory. J Neurosci.

[79] Palop JJ, Mucke L (2016). Network abnormalities and interneuron dysfunction in Alzheimer disease. Nat Rev Neurosci.

[80] Sasaguri H, Nilsson P, Hashimoto S (2017). APP mouse models for Alzheimer's disease preclinical studies. EMBO J.

[81] Bezzina C, Verret L, Juan C (2015). Early onset of hypersynchronous network activity and expression of a marker of chronic seizures in the Tg2576 mouse model of Alzheimer's disease. PLoS ONE.

[82] Busche MA, Konnerth A (2016). Impairments of neural circuit function in Alzheimer's disease. Philos T Roy Soc B.

[83] Ramírez-Toraño F, García-Alba J, Bruña R (2021). Hypersynchronized Magnetoencephalography Brain Networks in Patients with Mild Cognitive Impairment and Alzheimer's Disease in down Syndrome. Brain Connect.

[84] Noebels J (2011). A perfect storm: Converging paths of epilepsy and Alzheimer's dementia intersect in the hippocampal formation. Epilepsia.

[85] Yassa MA, Stark SM, Bakker A (2010). High-resolution structural and functional MRI of hippocampal CA3 and dentate gyrus in patients with amnestic Mild Cognitive Impairment. NeuroImage.

[86] Lam AD, Deck G, Goldman A (2017). Silent hippocampal seizures and spikes identified by foramen ovale electrodes in Alzheimer's disease. Nat Med.

[87] Wilson IA, Gallagher M, Eichenbaum H (2006). Neurocognitive aging: prior memories hinder new hippocampal encoding. Trends Neurosci.

[88] Leppik IE, Birnbaum AK (2010). Epilepsy in the Elderly. Ann NY Acad Sci.

[89] Olafsson E, Ludvigsson P, Gudmundsson G (2005). Incidence of unprovoked seizures and epilepsy in Iceland and assessment of the epilepsy syndrome classification: a prospective study. Lancet Neurol.

[90] Liu D, Lu H, Stein E (2018). Brain regional synchronous activity predicts tauopathy in 3×TgAD mice. Neurobiol Aging.

[91] Jacob L, Bohlken J, Schmitz B (2019). Incidence of epilepsy and associated factors in elderly patients in Germany. Epilepsy Behav.

[92] Koh MT, Haberman RP, Foti S (2010). Treatment Strategies Targeting Excess Hippocampal Activity Benefit Aged Rats with Cognitive Impairment. Neuropsychopharmacology.

[93] Sanchez PE, Zhu L, Verret L (2012). Levetiracetam suppresses neuronal network dysfunction and reverses synaptic and cognitive deficits in an Alzheimer's disease model. P Natl Acad Sci.

[94] Wolf P (2017). Reflex epileptic mechanisms in humans: Lessons about natural ictogenesis. Epilepsy Behav.

[95] Wieser HG (1998). Seizure induction in reflex seizures and reflex epilepsy. Adv Neurol.

[96] Arslan Y, Yilmaz Z, Mülayim S (2013). Eating Epilepsy After Resection of Frontal Meningioma: A Case Report. Arch Epilepsy.

[97] Ferlazzo E, Zifkin BG, Andermann E (2005). Cortical triggers in generalized reflex seizures and epilepsies. Brain.

[98] Szűcs A, Rosdy B, Kelemen A (2019). Reflex seizure triggering: Learning about seizure producing systems. Seizure.

[99] Falip M, Rodriguez-Bel L, Castañer S (2018). Musicogenic reflex seizures in epilepsy with glutamic acid decarbocylase antibodies. Acta Neurol Scand.

[100] Gelisse P, Thomas P, Padovani R (2003). Ictal SPECT in a case of pure musicogenic epilepsy. Epileptic Disord.

[101] Jallon P, Heraut LA, Vanelle JM (1989). Musicogenic epilepsy. Reflex Seizures and Reflex Epilepsies, Editions Médicine et Hygiène, Geneva.

[102] Tezer FI, Bilginer B, Oguz KK (2014). Musicogenic and spontaneous seizures: EEG analyses with hippocampal depth electrodes. Epileptic Disord.

[103] Luczak A, McNaughton BL, Harris KD (2015). Packet-based communication in the cortex. Nat Rev Neurosci.

[104] Luczak A, Barthó P, Harris KD (2009). Spontaneous Events Outline the Realm of Possible Sensory Responses in Neocortical Populations. Neuron.

[105] Bortel A, Yao ZS, Shmuel A (2019). A rat model of somatosensory-evoked reflex seizures induced by peripheral stimulation. Epilepsy Res.

[106] Boly M, Jones B, Findlay G (2017). Altered sleep homeostasis correlates with cognitive impairment in patients with focal epilepsy. Brain.

[107] Sitnikova E, Grubov V, Hramov AE (2020). Slow-wave activity preceding the onset of 10–15-Hz sleep spindles and 5–9-Hz oscillations in electroencephalograms in rats with and without absence seizures. J Sleep Res.

[108] van Luijtelaar G, Hramov A, Sitnikova E (2011). Spike–wave discharges in WAG/Rij rats are preceded by delta and theta precursor activity in cortex and thalamus. Clin Neurophysiol.

[109] Silva BA, Astori S, Burns AM (2021). A thalamo-amygdalar circuit underlying the extinction of remote fear memories. Nat Neurosci.

[110] Genzel L, Dragoi G, Frank L (2020). A consensus statement: defining terms for reactivation analysis. Philos T Roy Soc B.

[111] Nader K, Schafe GE, Le Doux JE (2000). Fear memories require protein synthesis in the amygdala for reconsolidation after retrieval. Nature.

[112] Winters BD, Tucci MC, DaCosta-Furtado M (2009). Older and stronger object memories are selectively destabilized by reactivation in the presence of new information. Learn Memory.

[113] Simon KCNS, Gómez RL, Nadel L (2018). Losing memories during sleep after targeted memory reactivation. Neurobiol Learn Mem.

[114] Brunet A, Saumier D, Liu A (2018). Reduction of PTSD Symptoms With Pre-Reactivation Propranolol Therapy: A Randomized Controlled Trial. Am J Psychiat.

[115] Schwabe L, Nader K, Wolf OT (2012). Neural Signature of Reconsolidation Impairments by Propranolol in Humans. Biol Psychiatry.

[116] Cahill L, Pham CA, Setlow B (2000). Impaired Memory Consolidation in Rats Produced with β-Adrenergic Blockade. Neurobiol Learn Mem.

[117] Soeter M, Kindt M (2015). An Abrupt Transformation of Phobic Behavior After a Post-Retrieval Amnesic Agent. Biol Psychiatry.

[118] LaBar KS, Cabeza R (2006). Cognitive neuroscience of emotional memory. Nat Rev Neurosci.

[119] Liang KC, Juler RG, McGaugh JL (1986). Modulating effects of posttraining epinephrine on memory: Involvement of the amygdala noradrenergic system. Brain Res.

[120] Dunsmoor JE, Niv Y, Daw N (2015). Rethinking Extinction. Neuron.

[121] Blundell J, Kouser M, Powell CM (2008). Systemic inhibition of mammalian target of rapamycin inhibits fear memory reconsolidation. Neurobiol Learn Mem.

[122] Galanopoulou AS, Buckmaster PS, Staley KJ (2012). Identification of new epilepsy treatments: Issues in preclinical methodology. Epilepsia.

[123] González Otárula KA, von Ellenrieder N, Cuello-Oderiz C (2019). High-Frequency Oscillation Networks and Surgical Outcome in Adult Focal Epilepsy. Ann Neurol.

[124] Roullet P, Vaiva G, Véry E (2021). Traumatic memory reactivation with or without propranolol for PTSD and comorbid MD symptoms: a randomised clinical trial. Neuropsychopharmacology.

[125] Trenite DGAK-N, DiVentura BD, Pollard JR (2019). Suppression of the photoparoxysmal response in photosensitive epilepsy with cenobamate (YKP3089). Neurology.

[126] Schjetnan AG, Luczak A (2011). Recording large-scale neuronal ensembles with silicon probes in the anesthetized rat. JoVE (Journal of Visualized Experiments).

[127] Luczak A, Narayanan NS (2005). Spectral representation—analyzing single-unit activity in extracellularly recorded neuronal data without spike sorting. J Neurosci Meth.

[128] Ryait H, Bermudez-Contreras E, Harvey M (2019). Data-driven analyses of motor impairments in animal models of neurological disorders. PLoS Biology.

[129] Luczak A, McNaughton BL, Kubo Y (2022). Neurons learn by predicting future activity. Nat Mach Intell.

[130] Chalmers E, Contreras EB, Robertson B, Luczak A, Gruber A (2017). Learning to predict consequences as a method of knowledge transfer in reinforcement learning. IEEE T Neural Network Learn Systems.

